# Integration of risk mitigation programmes in stunting prevention efforts for early childhood

**DOI:** 10.4102/jamba.v17i1.1832

**Published:** 2025-08-26

**Authors:** Syahria A. Sakti, Latifah Putranti, Yulian A. Suminar, Romaito Dongoran

**Affiliations:** 1 Department of Early Childhood Education, Faculty of Teacher Training and Education, Universitas PGRI Yogyakarta, Yogyakarta, Indonesia; 2 Department of Management, Faculty of Business and Law, Universitas PGRI Yogyakarta, Yogyakarta, Indonesia; 3 Department of Special Education, Faculty of Teacher Training and Education, Universitas PGRI Yogyakarta, Yogyakarta, Indonesia

**Keywords:** stunting prevention, early childhood, risk mitigation, integrated programs, public health 5 intervention, child nutrition, health policy, community-based approach, preventive strategies

## Abstract

Stunting in early childhood remains a pressing public health issue in Gunungkidul Regency, Yogyakarta, Indonesia, which also faces frequent environmental challenges such as droughts. These challenges necessitate innovative approaches to ensure children’s health and resilience. The purpose of this research is to examine the integration of mitigation programmes with efforts to combat stunting in early infancy, addressing the dual challenge of enhancing resilience while reducing stunting prevalence. This study employs a qualitative case study approach. Participants include key stakeholders such as health staff, government agencies, and parents of stunted children. The research findings reveal that integrating mitigation programmes can significantly enhance children’s resilience to adverse conditions and prevent stunting by building community capacity, educating the public on preparedness and nutrition, and improving access to health services. The study identifies significant barriers, such as difficulties in sector coordination, low public awareness of the relationship between environmental risks and stunting, and limited resources. The findings have important policy and practical implications. Policymakers should prioritise cross-sector collaboration, allocate sufficient resources, and strengthen public awareness campaigns to ensure the effectiveness of integrated programmes. Practical interventions could include targeted training for healthcare providers and educators, community engagement in nutrition initiatives, and evidence-based decision-making to address these intertwined challenges comprehensively.

**Contribution:**

Exploration of the intersection between risk mitigation efforts and stunting prevention in early childhood provides valuable insights into how integrated programmes can enhance child resilience and prevent stunting in vulnerable communities. These findings offer actionable recommendations for improving policies and programme design in Indonesia while also serving as a reference for regions facing similar challenges globally, thereby contributing to broader discussions on public health and community resilience.

## Introduction

Stunting, defined as impaired growth and development in children because of chronic malnutrition, is a pressing global public health issue. According to United Nations Children’s Fund (UNICEF), approximately 22% of children under the age of five worldwide were stunted in 2022, with the highest prevalence in sub-Saharan Africa and South Asia (Milman et al. 2005). For instance, in Ethiopia, over 37% of children under five are stunted (Ahmed et al. 2021). This condition results from prolonged nutritional deficiencies, particularly the lack of essential nutrients such as protein, iron, and zinc. Stunting is further exacerbated by poor sanitation and hygiene practices, which increase the risk of infections such as diarrhoea, leading to nutrient loss. Maternal malnutrition also contributes significantly, as undernourished mothers are more likely to give birth to low-birth-weight infants at higher risk of stunting (Fidian et al. 2022). Social determinants, including poverty, limited education, and inadequate access to healthcare, compound these challenges (Angraini 2024).

In Gunungkidul Regency, Yogyakarta Province, the Karangmojo community faces a particularly high prevalence of stunting. According to the 2022 report by the Yogyakarta Spesial Region (DIY) National Population and Family Planning Agency (BKKBN), the stunting rate in this region stands at 35%, highlighting a pressing need for targeted interventions. The high prevalence of stunting in Karangmojo is compounded by the region’s vulnerability to natural hazards, including earthquakes, tsunamis, and droughts. These disasters disrupt access to clean water, healthcare, and food supplies, intensifying the underlying risk factors for stunting. Disaster-induced food insecurity, for example, arises from droughts and floods that disrupt agriculture and food supply chains, leading to acute and chronic food shortages. Climate change aggravates these risks by amplifying extreme weather events that threaten food systems and livelihoods, worsening malnutrition and stunting rates (Puspita, Soesanto & Muhammad 2019). Disasters also damage water and sanitation infrastructure, increasing exposure to waterborne diseases such as diarrhoea, a major contributor to stunting (Gaire et al. 2016). Vulnerable households, particularly in low-income communities, are disproportionately affected due to limited resilience and coping mechanisms (Ayuni & Arsil 2021).

Children suffering from stunting often exhibit reduced physical strength, lower immunity, and diminished recovery capacities in the aftermath of disasters (Kudus & Dewi 2020). This interconnection underscores the importance of addressing stunting and disaster risks in tandem. Despite isolated efforts to tackle these challenges, there remains a gap in integrated programme planning that holistically addresses both concerns. This study focuses on Karangmojo in Gunungkidul, a region where the dual burden of stunting and disaster risks converges, creating a compelling need for innovative strategies. The rationale for this study lies in its potential to identify synergies between stunting prevention and disaster mitigation programmes, thus addressing two critical challenges simultaneously. By leveraging the combined expertise of multiple stakeholders, including local government, non-governmental organisations, and community members, integrated programmes could yield sustainable solutions for child welfare in disaster-prone regions.

In addressing the dual challenge of stunting and environmental vulnerabilities in Gunungkidul Regency, integrating relevant theoretical frameworks enhances the study’s depth and applicability. The Sustainable Livelihoods Framework (SLF) underscores how environmental shocks, such as droughts, disrupt livelihoods, exacerbate food insecurity, and heighten stunting risks, emphasising the role of human, natural, financial, and social capital in resilience-building (Nikolakis & Grafton 2015). The UNICEF Conceptual Framework of Malnutrition provides a structured approach to identifying the immediate, underlying, and basic causes of stunting, linking disaster-induced food insecurity and disease outbreaks to nutritional deficits (Black, Lutter & Trude 2020). Furthermore, the Sendai Framework for Disaster Risk Reduction (2015–2030) advocates for integrating nutrition security into disaster risk management to mitigate the long-term effects of environmental hazards on child development (United Nations Office for Disaster Risk Reduction [UNDRR] [Wahlstrom 2017]). In addition, Community-Based Disaster Risk Management (CBDRM) highlights the importance of local participation in formulating nutrition-sensitive disaster resilience strategies, fostering community-led solutions to the issue of stunting (Bozzola et al. 2022). Integrating these frameworks into mitigation programmes ensures a holistic approach to strengthening resilience and reducing stunting prevalence in disaster-prone areas.

While there is extensive research on the causes and impacts of stunting and on risk reduction efforts, few studies explore the integration of these two critical areas. Research by Supriyanto (2022) highlights how limited access to nutritious food, poverty, and unbalanced dietary patterns contribute to stunting in rural Indonesia. Similarly, Picauly (2021) emphasises the importance of education and healthcare access in mitigating stunting. However, various external factors could cause these vulnerabilities by disrupting food security, health infrastructure, and community resilience. Febrian (2021) argues that merging risk reduction strategies with nutrition-focused programmes can strengthen children’s resilience, while Fitrauni (2019) demonstrates that comprehensive risk management efforts reduce stunting risks and enhance community health outcomes. This study aims to address this gap by examining the integration of risk mitigation programmes into stunting prevention efforts in Karangmojo, Gunungkidul Regency. An effective stunting prevention programme can help children build resilience by improving their physical health, enhancing their nutritional status, and strengthening their capacity to recover and thrive in the face of calamities. This resilience encompasses their ability to maintain adequate growth, cognitive development, and immune response despite the challenges posed by disasters (Edwards, Gray & Borja 2020).The integration of catastrophe mitigation programmes and stunting prevention efforts might concentrate on increasing children’s resilience. These programmes may include disaster mitigation and nutrition education and awareness activities, as well as the provision of nutritious food, meeting the fundamental requirements of children, and disaster recovery. To identify and evaluate children’s needs and build integrated programmes, multiple stakeholders must work together closely (Prambudi 2018). Meeting children’s fundamental needs is also a priority in these programmes. Children in disaster zones frequently lack access to adequate water, sanitation, healthcare, and education (Mallick et al. 2012). As a result, these programmes work to ensure that children have sufficient access to such facilities and services. This involves providing clean water, safe and appropriate bathrooms, basic healthcare, and access to high-quality education.

Addressing stunting not only improves children’s overall health but also enhances community resilience (Datar et al. 2013). By ensuring children achieve optimal physical and cognitive development, communities become better equipped to face various challenges that may arise. Healthy, well-nourished children are more likely to adapt and recover effectively in difficult situations, contributing to the long-term strength and stability of the population. In addition, stunting prevention programmes help children achieve good nutrition, resulting in stronger immunity against illnesses and infections (Acosta 2012). Programmes aimed at reducing stunting play a crucial role in ensuring the well-being of children and communities. While the development of resilient infrastructure, such as fortified health and education facilities, is essential, findings indicate that such infrastructure is still under development or remains limited in many areas. Strengthening infrastructure to ensure continued access to essential health and nutrition services is necessary to support stunting prevention efforts.

The successful implementation of these programmes requires collaboration among multiple stakeholders, including the government, international organisations, non-governmental organisations, and local communities (Maskrey 2011). These stakeholders work together to design and implement coordinated initiatives, sharing expertise, resources, and experiences to address challenges effectively.

Integrating mitigation programmes and efforts to combat stunting in early childhood is essential for children’s well-being and development (Bartlett & Steber 2019). Environmental and social hazards can significantly impact children, leading to physical and mental health challenges as well as disruptions in their education. Early childhood education institutions are particularly vulnerable to adverse events, while young children often lack awareness and capacity for risk mitigation during natural hazards. Schools also lack adequate preparedness programmes and educational tools, highlighting the need for effective and practical learning media to teach young students about risk reduction. According to Shonkoff (2009), exposure to challenging situations can affect children’s mental health and behavioural responses, influencing their physical well-being, social vulnerability, and overall development. In addition, sociodemographic factors, institutional roles, and broader socio-ecological contexts play a crucial role in shaping children’s experiences and outcomes. Therefore, incorporating risk mitigation strategies into early childhood education is vital to ensuring that young children receive appropriate support and knowledge in facing potential hazards.

This research focuses on the interconnected dimensions of child development and health, providing actionable insights for integrating stunting prevention with local resilience-building efforts. The findings aim to guide stakeholders in designing comprehensive and sustainable programmes tailored to the specific needs of the people of Gunungkidul region. This integration can yield dual benefits by enhancing children’s resilience to various challenges while contributing to stunting prevention (Masten 2021). It is hoped that this research will significantly contribute to the development of more effective policies and programme planning to address the problem of stunting in Gunungkidul, Yogyakarta. In this light, the study offers an innovative approach by merging efforts to reduce stunting with community resilience initiatives. This research intends to provide a more comprehensive understanding of the programme’s challenges, successes, and potential for integration. The study will address the following questions:

- *What strategies are currently being implemented to address stunting in early childhood in Karangmojo, Gunungkidul District, Yogyakarta?*
- *What are the key challenges and enabling factors in integrating resilience-building programmes with stunting prevention efforts in Karangmojo, Gunungkidul District, Yogyakarta?*
- *What recommendations can enhance the integration of resilience-building programmes and stunting prevention initiatives to ensure sustainability in Karangmojo, Gunungkidul District, Yogyakarta?*

## Research methods and design

This study employs a qualitative approach, using a case study method. The qualitative data analysis approach in this research follows an interactive model encompassing three key stages: data reduction, data presentation, and drawing conclusions (Lincoln & Guba 2000). The qualitative technique was chosen as the study aims to gain a thorough understanding of the integration of mitigation programmes and efforts to combat stunting in early infancy in Gunungkidul, along with the supporting factors and challenges involved. Gunungkidul was selected as the research location because of the fact that it has the highest prevalence rate of stunting among districts in Yogyakarta. Karangmojo was selected as the study area because of its consistently high prevalence of stunting cases within Gunungkidul, making it a critical location for examining the effectiveness of integrated mitigation and stunting prevention programmes. A qualitative analysis was conducted using data collected through interviews, observations, and document analysis to gain a comprehensive understanding of programme implementation. Observations focused on how mitigation and stunting prevention efforts were carried out in Karangmojo, including the roles and activities of health workers, the level of community engagement during programme implementation, and the availability and accessibility of essential resources such as clean water, nutrition, and healthcare services.

This study includes a sample size of eight respondents, consisting of policymakers, health workers, and parents from Karangmojo, Gunungkidul. The respondents are as follows: three policymakers (local government officials involved in health and policy implementation), three health workers (community health centre staff and field officers), and two parents of children identified as stunted, representing the community perspective (Table 1). Data validity is ensured through data triangulation, which involves comparing findings from multiple data sources (interviews, observations, and document analysis) to verify consistency and reliability. Based on the findings of the data analysis, conclusions were drawn regarding the implementation of mitigation programmes and stunting prevention efforts, the factors that support or hinder programme integration, and the effectiveness of these integrated programmes in enhancing children’s resilience and reducing stunting rates in Gunungkidul.

**TABLE 1:** Respondent profiles.

Initial	Gender	Age (years)	Occupation
R1	Male	57	Village head
R2	Male	45	Health workers
R3	Female	52	Public health
R4	Male	40	Social services
R5	Female	23	Parent
R6	Female	21	Parent
R7	Female	20	Parent
R8	Female	20	Parent

### Ethical considerations

Ethical clearance to conduct this study was obtained from the Universitas PGRI Yogyakarta Ethics Committee/Institutional Review Board (No: 300/BAP-LPPM/I/2025).

## Results

### Efforts to overcome stunting in early childhood in Gunungkidul, Yogyakarta

**FIGURE 1:**
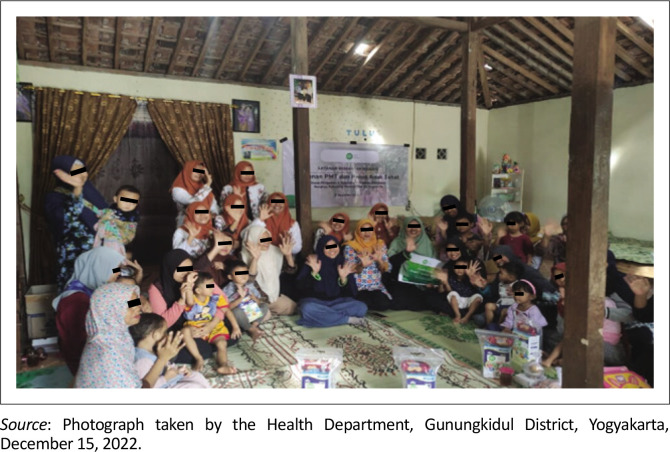
Community engagement in stunting prevention.

Interviews with study participants revealed that multiple stakeholders have undertaken initiatives to address stunting in the Karangmojo, Gunungkidul area. The integration efforts developed to tackle the issue of stunting involve multiple parties, including local government officials, community health workers, and parents of affected children. Local government agencies play a key role in formulating and implementing policies aimed at reducing stunting rates, while healthcare professionals focus on early detection and intervention efforts in high-prevalence areas. Civil society organisations and local community groups also contribute to these efforts by raising awareness and facilitating access to essential resources such as nutrition and clean water (see Figure 1). One of the policymakers interviewed emphasised the government’s commitment to addressing the issue:

‘… the local government has made various efforts to overcome the stunting problem. First of all, we have enhanced the availability and quality of health care in this area. We have improved health infrastructure by constructing and upgrading community health centres and posyandu. We are also strengthening the training of medical workers in recognising and treating stunting in children …’ (R1)

In addition to efforts by the local government, the health department has taken proactive measures to address stunting through early detection efforts. These include gathering data from the community to identify key factors contributing to stunting. One health worker stated:

‘… based on my research, there are several factors that contribute to stunting in Kulonprogo. These factors include lack of nutrition in pregnant women and babies, low levels of education and awareness of mothers about good nutrition, limited access to health care, and poor sanitation. All of these factors interact and can cause stunting in children …’ (R2)

**FIGURE 2:**
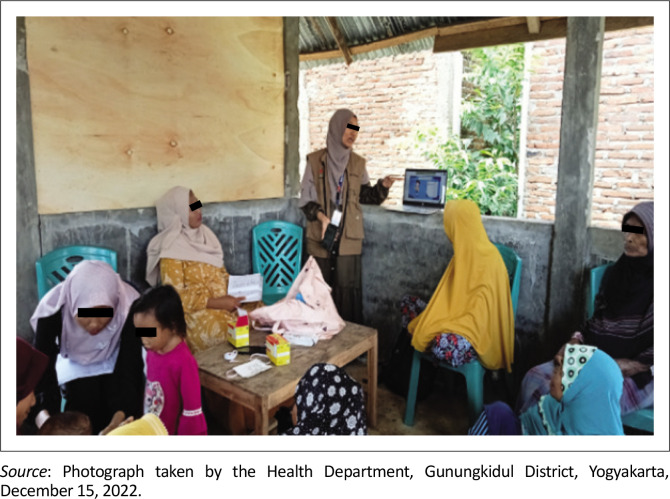
Engagement of a team of expert nutritionists from the National Hospital.

Findings from observations and interviews indicate that integrating mitigation programmes with stunting prevention efforts can significantly enhance children’s resilience while addressing the root causes of stunting. Observations of community-based programmes in Karangmojo revealed that areas with improved sanitation infrastructure and better access to clean water had lower reported cases of stunting. Furthermore, direct observation of health education sessions showed that parents who participated in nutrition and hygiene workshops demonstrated increased awareness of the link between poor sanitation and malnutrition (see Figure 2). Interviews with health workers reinforced these observations. For example, a community health worker observed that:

‘… many cases of stunting in Karangmojo are linked to inadequate access to clean water and poor hygiene practices, which also increase vulnerability to environmental hazards.’ (R5)

Another health worker (Respondent 6) highlighted that educational programmes on proper waste disposal and household hygiene have reduced both stunting cases and health risks during environmental challenges, such as seasonal water shortages.

**FIGURE 3:**
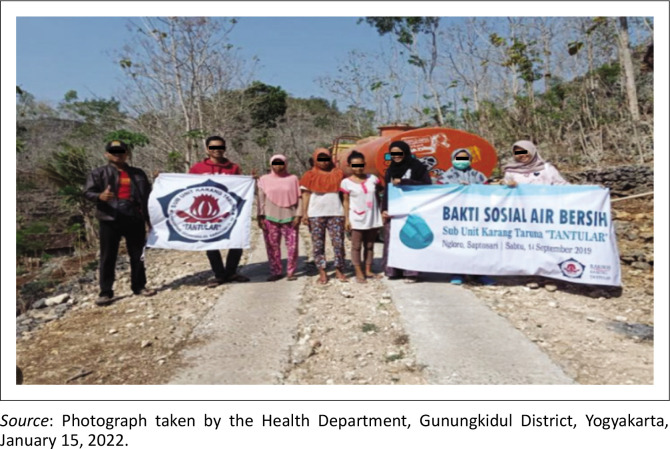
Karangmojo Gunungkidul research site, an area with a clean water crisis during the dry season.

The findings from observations and interviews indicate that community-level training programmes play a crucial role in fostering resilience. These training sessions provide practical steps for household preparedness and nutrition education, empowering families to enhance both child health and overall readiness (see Figure 3). Observations revealed that local initiatives focus on improving nutritional awareness, sanitation, and hygiene as part of an integrated effort to address stunting and other related challenges. Interview data further support these findings. Regarding this, one policymaker explained the following:

‘We have launched an educational program to increase nutritional awareness among pregnant women and toddlers. We collaborate with health institutions and non-governmental organizations to promote good eating habits, provide nutritional supplements, and assist with child care. Additionally, we have implemented sanitation and hygiene programs. We construct proper sanitary facilities and ensure community access to clean water. We also educate the public on the importance of personal hygiene, good sanitation, and waste management.’ (R3)

One of the main advantages of programme integration is increased resilience in children to cope with the effects of disasters. Young children are most vulnerable to the effects of disasters, both physically and emotionally. In catastrophe scenarios, access to adequate food and health services may be disrupted, increasing the risk of stunting. A complete strategy for meeting children’s needs, even in emergency situations, can be achieved by combining disaster mitigation and stunting prevention programmes. An integrated approach combines disaster mitigation and stunting control initiatives in a single comprehensive framework. This can include incorporating activities such as providing healthy food, monitoring children’s growth, boosting access to health services, constructing disaster-resistant infrastructure, and raising community knowledge and understanding of disaster risks and stunting.

Furthermore, an integrated approach entails a cross-sectoral approach and active participation from all important stakeholders, such as the government, non-governmental organisations, educational institutions, communities, and families (Nwosu & Ataguba 2020). This partnership provides for synergy in disaster mitigation and stunting control efforts, as well as ensuring that policies and programmes are appropriate for local requirements and contexts. To obtain a clear image of the conditions and demands confronting the integrated efforts of the above-mentioned partnership, a strategy is devised to encourage active participation from the community, which serves as a social capital for disaster mitigation in an area:

‘… regular monitoring and evaluation of the programs we have implemented. We conduct regular nutrition surveys to track stunting levels and progress in this region. Aside from that, we gather information on community participation in nutrition and sanitation programmes. The evaluation results demonstrate that there has been an increase in community understanding of the necessity of healthy nutrition and sanitation. We have witnessed a rise in the number of women taking part in nutrition education programmes, as well as increased access to sufficient sanitary facilities. While there are still hurdles to face, we are certain that the efforts we have taken have resulted in significant progress.” We also plan to increase collaboration with the private sector, non-governmental organisations (NGOs) and other institutions to support stunting reduction programs …’ (R4)

Robust partnerships between various stakeholders can increase access to the resources, knowledge and support needed to effectively achieve stunting reduction goals. The study by Absori et al. (2022) underlines the need of collecting high-quality data and conducting frequent impact analyses of treatments. This is critical for assessing the effectiveness of the programme and identifying areas for improvement in efforts to combat stunting. Efforts to combat stunting in early infancy in Gunungkidul, Yogyakarta, emphasise the need of a multi-stakeholder strategy involving the government, community health centres, local communities, the private sector, and non-governmental organisations (NGOs). By combining knowledge from diverse research and field experience, it is believed that these initiatives would have a substantial positive influence on lowering stunting and enhancing the welfare of children in the area.

### Supporting factors and obstacles in the integration of risk mitigation programmes and stunting reduction efforts

**FIGURE 4:**
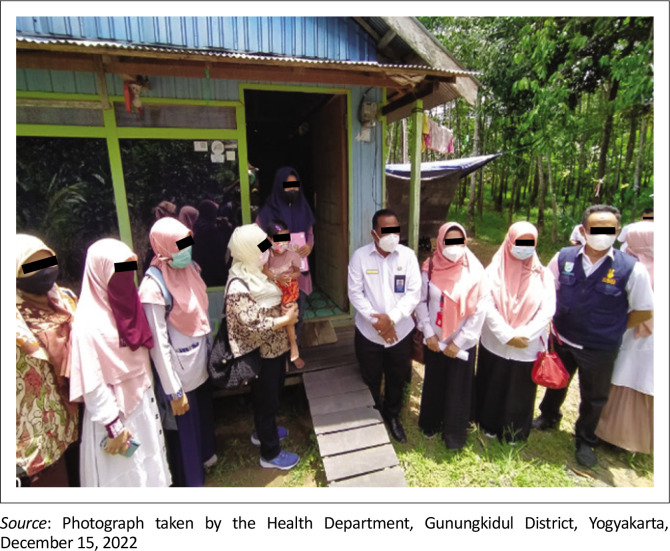
Patterns of partnerships built to overcome the problem of stunting.

Increasing community and local government knowledge of the link between catastrophes and stunting is critical to the programme’s inclusion. Understanding that post-disaster conditions can increase the risk of stunting motivates governments and communities to act (Aguayo & Menon 2016). The collaboration between the health authorities, disaster management, and development sectors is critical. Programme integration necessitates close coordination among all stakeholders, including the government, non-governmental organisations, and civil society. The emphasis is on community-based approaches. It is critical to encourage strong community participation in programme conception, implementation, and monitoring (see Figure 4). In doing so, local communities’ capability will be strengthened, ensuring the programme’s sustainability and relevance:

‘… sometimes, lack of effective communication between various stakeholders can hinder the implementation of these programs. Poor coordination between the health, disaster management and development sectors can lead to overlapping or duplication of efforts, as well as unclear roles and responsibilities of each party …’ (R5)

Several challenges exist in raising public awareness and understanding of the link between environmental hazards and stunting. Observations and interviews with respondents revealed that limited knowledge about the long-term impacts of such events on children’s nutritional status hampers efforts to integrate mitigation and stunting prevention programmes. This finding aligns with previous studies (Masten 2021), which emphasise that disruptions in food supply, access to clean water, and healthcare services can significantly contribute to malnutrition and stunting in young children. One policymaker highlighted this issue, stating the following:

‘Many communities are unaware that environmental hazards, such as droughts or floods, can directly affect children’s nutrition by disrupting access to food, clean water, and healthcare services. This is why we need stronger communication strategies to educate the public about these connections.’ (R3)

This perspective is consistent with the research by Spencer and Thompson (2024), which underscores the importance of public awareness in mitigating the indirect effects of environmental challenges on child health. Strengthening communication strategies and community engagement is therefore essential to ensuring the successful integration of programmes aimed at reducing stunting and improving the well-being of children.

Findings from this study align with Beal et al. (2018), who emphasise that effective information dissemination can enhance public awareness and motivate stakeholders to act. Local governments with a deeper understanding of the disaster–stunting link can develop targeted interventions. For instance, a health worker, noticed:

‘We have conducted community education programs to explain how poor sanitation and lack of clean water during disasters increase stunting risks. These efforts help communities see the urgency of both disaster preparedness and proper child nutrition.’ (R4)

The integration of health, disaster management, and development sectors was a recurring theme in interviews. A local government official, highlighted the benefits of cross-sector collaboration:

‘By involving various stakeholders – health workers, disaster management teams, and NGOs – we’ve been able to create programs that address both immediate disaster impacts and long-term stunting prevention. For example, disaster relief efforts now include nutritional supplements for children.’ (R5)

This approach aligns with the findings by Beal et al. (2018) and Patel et al. (2008), who argue that synergy between various sectors can amplify the effectiveness of interventions. Stakeholders, including government agencies, NGOs, and civil society groups, play a critical role in fostering collaboration to achieve shared goals of reducing stunting. Thus, the findings demonstrate that raising public awareness and enhancing cross-sector collaboration are crucial steps in addressing the interconnected challenges of stunting. These insights are derived from interviews, observations, and supporting literature, offering practical recommendations for policymakers and practitioners.

The connection between environmental challenges and stunting is often underrecognised, leading to gaps in the efforts to prevent malnutrition in children. Research by Beal et al. (2018) highlights that effective communication strategies are vital to raising public awareness. However, to fully understand this link, more rigorous education and outreach efforts are required, particularly in vulnerable regions such as Yogyakarta, where factors such as food insecurity and disruptions to health services pose significant risks. However, various external factors may contribute to these vulnerabilities by undermining food security, weakening health infrastructure, and reducing community resilience.

The findings of this study underscore the importance of local governments developing a comprehensive understanding of the link between environmental challenges and stunting to drive targeted interventions at both the policy and programmatic levels. Interviews with respondents revealed that collaboration between public health and related agencies is crucial for addressing these interconnected issues. For instance, a policymaker, observed:

‘By working together, health and disaster management teams can ensure that emergency responses include nutritional support for children while simultaneously building long-term resilience through community education and health initiatives.’ (R3)

This sentiment is echoed in observations of integrated programmes in Karangmojo, where health workers and response teams have aligned their efforts to combine preparedness activities with stunting prevention measures. For example, during drought relief initiatives, the distribution of clean water is accompanied by public education on hygiene and nutrition, as highlighted by a health worker:

‘When we provide clean water during droughts, we also use the opportunity to teach families about proper nutrition and sanitation practices to prevent stunting.’ (R5)

The integration of disaster mitigation and stunting prevention strategies reflects findings from Beal et al. (2018) and Patel et al. (2008), which show that cross-sector collaboration amplifies the impact of interventions. The study also found that aligning emergency response measures with long-term health programmes, such as those targeting children’s nutrition, can address immediate challenges while building resilience against future crises. These findings demonstrate the potential for synergy when health, management, and development sectors collaborate. The insights gained from respondents and observations provide a practical foundation for local governments to develop and implement integrated strategies that enhance children’s resilience while reducing stunting prevalence.

A case in point can be seen in Yogyakarta, where coordination between local governments, NGOs, and civil society organisations has proven essential. This collaboration facilitates the implementation of resilient health programmes and ensures continuity in nutritional support for affected children. By fostering multi-stakeholder partnerships, the response to both immediate impacts and the longer-term risk of stunting becomes more robust. Therefore, reducing stunting in children requires ongoing cooperation, strong political commitment, and the integration of resilience strategies into stunting prevention initiatives. This approach will create a more sustainable framework capable of protecting children’s health in times of crises.

### Recommendations for improving the integration of mitigation programmes and stunting prevention efforts

Local governments and related organisations must create integrated action plans to simultaneously address risk mitigation and stunting prevention. This involves mapping vulnerable locations, identifying risks, and developing comprehensive mitigation strategies. The findings from interviews with respondents reveal that collaborative efforts are already in place. A policymaker, highlighted:

‘We have built close coordination between related institutions, such as the Health Service, Education Service, and the Management Agency. We have integrated risk mitigation aspects into existing stunting prevention programs. Apart from that, we also provide training to teachers and medical staff regarding handling stunting in emergency situations.’ (R1)

The active involvement of local communities in the formulation, implementation, and monitoring of stunting reduction programmes is also under consideration. According to Hall et al. (2018), community participation in nutrition education activities, feeding practice training, and sanitation facility construction can improve programme efficacy and ensure the intervention’s long-term sustainability. Research by Tampy, Nugroho and Syuadzah (2020) found that intervention programmes backed by community health centres, such as supplementary feeding and nutrition education, have the potential to significantly reduce stunting prevalence. Strong relationships among various stakeholders can improve access to the resources, information, and support required to effectively meet stunting reduction goals. Based on the interviews, the study reveals that community involvement is a key strategy in integrating risk mitigation and stunting prevention programmes. A parent, emphasised the importance of community participation:

‘The government has also engaged communities in the creation and implementation of these programs, giving them a sense of ownership and responsibility for stunting prevention and risk management initiatives. Parents are informed and educated about mitigation strategies and the necessity of avoiding stunting.’ (R6)

In addition, policymakers acknowledged the role of research in enhancing programme effectiveness. A health worker, stated:

‘We greatly value the contribution of research in informing policy and strengthening the scientific basis of our programs. Data and evidence obtained from research help us make better decisions and increase the effectiveness of risk mitigation and stunting control programs.’ (R7)

Further insights were provided by a policymaker, who highlighted the necessity of central government support:

‘I would like to emphasize the necessity of central government assistance in providing appropriate human resources, funding, and infrastructure to enable the integration of this program. Furthermore, research institutions can continue to perform comprehensive and long-term research to aid regional development. Other relevant parties, such as non-governmental organizations and civil society, must also actively participate in the implementation and monitoring of these programs.’ (R8)

These statements underscore the collaborative nature of the initiatives, involving multiple stakeholders such as government agencies, research institutions, NGOs, and civil society (see Figure 3). The findings reflect a shared commitment to leveraging resources, research, and community engagement to ensure the success and sustainability of these integrated programmes. The integration of risk mitigation programmes with stunting prevention activities is a strategic step towards increasing community resilience to these challenges. By adopting an integrated approach, collaborating across sectors, and demonstrating strong commitment among various stakeholders, we can create a safer, healthier, and more sustainable environment for future generations. Through these efforts, communities will become more resilient in the face of emergencies and better equipped to solve the problem of stunting, allowing future generations to grow and develop to their full potential. Investments in resilient health and sanitation facilities can enhance response times while simultaneously strengthening efforts to combat stunting. Building good health centres, as well as providing access to clean water and sanitation, would help to improve children’s nutritional status significantly. Education and training programmes aimed at local communities, particularly pregnant and breastfeeding women, can raise awareness of the importance of proper nutrition and healthy living habits. This can take the form of training seminars, outreach efforts, or community forums.

## Conclusion

The research provides insight into the need to integrate disaster mitigation programmes with stunting prevention initiatives in early childhood. In this study, it was found that the integration of mitigation programmes and stunting prevention efforts in Gunungkidul has resulted in several concrete steps. The involvement of various related institutions, such as the Health Service, Education Service, and Management Agency, is key in achieving holistic integration. This programme’s effectiveness is partly dependent on community involvement and increased public awareness. However, this study also highlights some of the difficulties encountered in integrating these programmes. The primary obstacles identified were a lack of human resources and a limited budget. Central government assistance is required to provide appropriate resources and empower research institutions to continue conducting in-depth and sustained research. In this scenario, central government assistance and collaboration among research institutions, local governments, and the community are essential for generating favourable and long-term results. Evidence from this research indicates that mitigation measures, such as creating resilient infrastructure and community education, can directly support stunting prevention programmes by ensuring continuous access to health and nutritional services during crises. Thus, integrating mitigation programmes with efforts to combat stunting in early childhood in Gunungkidul Yogyakarta has the potential to significantly improve children’s quality of life while also lowering the risk of negative consequences.

### Recommendations

The findings from this study inform several recommendations to address the dual challenges of stunting prevention and health risk mitigation in Gunungkidul:

- Expand programme scope and target areas: Respondents emphasised the need to scale up integrated programmes, particularly in high-risk areas such as Karangmojo. This expansion should prioritise communities with limited access to healthcare, education, and essential support resources. Document analysis of local government reports on vulnerable zones and stunting prevalence supports this approach by identifying specific areas requiring intervention.
- Strengthen cross-sector coordination: Based on interviews with policymakers and health workers, it was revealed that the integration of stunting prevention programmes with broader health and social initiatives requires greater collaboration between government agencies (e.g. Health Services, Education Services, and Community Development Agencies), NGOs, and civil society organisations. This is consistent with the literature, which highlights the effectiveness of cross-sector collaboration in achieving shared goals.
- Enhance community awareness: Respondents, including parents and health workers, emphasised the need to increase public awareness of the factors contributing to stunting. This involves delivering educational campaigns at the community level to promote preparedness and proper nutrition. Document analysis revealed that existing programmes do not sufficiently address the intersection of these issues, indicating a gap that can be filled through targeted education.
- Allocate adequate resources: Policymakers and health workers highlighted the necessity of adequate funding, infrastructure, and human resources to support programme implementation. This includes training medical staff and educators on handling stunting-related health challenges, as mentioned in interviews. Document analysis of government budgets and policies further suggests that resource allocation for integrated programmes remains limited and requires prioritisation of vulnerable groups. Gunungkidul, with its vulnerability to environmental risks and high stunting prevalence, presents a unique context where programme integration can deliver significant benefits. By addressing gaps in coordination, awareness, and resources, the effectiveness of these initiatives can be enhanced, ensuring better outcomes for children’s health and overall community well-being.
